# Comprehensive Chemometric and Chromatographic Investigation of Lipophilicity of Biologically Active Androstane-3-Oxime Derivatives Across Diverse UHPLC Systems

**DOI:** 10.3390/ph18121778

**Published:** 2025-11-23

**Authors:** Strahinja Z. Kovačević, Milica Ž. Karadžić Banjac, Sanja O. Podunavac-Kuzmanović, Jasmina S. Anojčić, Jovana J. Ajduković

**Affiliations:** 1 Department of Applied and Engineering Chemistry, Faculty of Technology Novi Sad, University of Novi Sad, Bulevar cara Lazara 1, 21000 Novi Sad, Serbia; strahko@uns.ac.rs (S.Z.K.); sanya.podunavac@uns.ac.rs (S.O.P.-K.); 2 Department of Chemistry, Biochemistry and Environmental Protection, Faculty of Sciences, University of Novi Sad, Trg Dositeja Obradovića 3, 21000 Novi Sad, Serbia; jasmina.anojcic@dh.uns.ac.rs (J.S.A.); jovana.ajdukovic@dh.uns.ac.rs (J.J.A.)

**Keywords:** artificial neural networks, biologically active molecules, chemometrics, chromatography, lipophilicity, small molecules, steroids

## Abstract

**Background/Objectives**: Previously reported analyses show that some androstane-3-oxime derivatives with picolyl and picolinylidene functional groups possess a significant anticancer activity towards various cancer cell lines. These findings suggest that these compounds have prominent biological potential and represent a good basis for further research. The present study aims to determine their anisotropic lipophilicity as a physicochemical parameter relevant to both prediction of chromatographic behavior and biological activity. **Methods**: Anisotropic lipophilicity was determined using reversed-phase ultra-high performance liquid chromatography (RP-UHPLC) systems equipped with three stationary phases (C18, C8 and phenyl) and three mobile phases composed of water with different modifiers (methanol, acetonitrile and a methanol-acetonitrile mixture). Capacity factors (log*k*) were obtained for all compounds across the chromatographic systems to describe their behavior in anisotropic environments. Chemometric analyses were performed using linear pattern recognition techniques (PRT), such as hierarchical cluster analysis (HCA) and principal component analysis (PCA), and non-linear clustering based on artificial neural networks (CANN). **Results**: The experimentally determined chromatographic parameters were correlated with in silico lipophilicity values (log*P*). This comparison allowed for examination of the concordance between experimental and computed data for the series of androstanes. The chemometric analysis resulted in models that provided an overview of the grouping of compounds in the space of the determined chromatographic parameters. **Conclusions**: The results demonstrate strong agreement between experimental and computational lipophilicity parameters. This very good data fit provides a reliable foundation for further studies exploring the relationships between lipophilicity and biological activity of the studied androstane derivatives.

## 1. Introduction

Steroidal derivatives are generally quite potent and safe biologically active compounds expressing various biological effects. Some naturally occurring steroidal derivatives, such as those from *Sarcococca* plants, have antitumor, hepatoprotective and immunosuppressive activity [1,2] Another derivative is sarsasapogenin, a natural steroidal sapogenin with anti-inflammatory, antidiabetic and neuroprotective activity [3,4,5]. Besides the plethora of natural steroids, the synthesis of novel steroidal derivatives with desirable biological activities is being continuously pursued to expand therapeutic potential, improve pharmacokinetic profiles and overcome limitations associated with natural analogues [6,7,8]. The synthesis of new androstane derivatives is driven by the need to refine and expand the biological potential of steroidal scaffolds beyond their natural limitations [9,10,11]. While the androstane core offers a framework for interaction with various biological targets, subtle structural modifications can significantly alter pharmacological profiles, enabling improved selectivity, potency and metabolic stability. Novel *O*-alkylated oxyimino androst-4-ene compounds, derivatized with different alkylaminoethyl side chains, were synthesized based on the recognition that steroidal oximes possess a diverse spectrum of biological activities, including pronounced antimicrobial, anticancer and antioxidant effects [12].

The physicochemical properties of novel biologically active compounds, including lipophilicity, solubility, polarizability, molar refractivity and so on, must be carefully evaluated to gain insight into their behavior within biological systems, their affinities for diverse environments and their potential interactions with other biologically relevant molecules. Chromatographic lipophilicity is an experimentally accessible measure of how different compounds interact with hydrophobic environments, which is crucial for predicting their behavior in biological systems [13,14,15]. By analyzing retention parameters in reversed-phase high performance liquid chromatography (RP-HPLC), the relative lipophilicity of steroid derivatives can be estimated and correlated with pharmacokinetic properties such as membrane permeability and bioavailability [15,16]. This approach also enables fine-tuning of molecular design, guiding the synthesis of steroidal agents with optimized therapeutic profiles. The chromatographic parameters obtained in different chromatographic systems can be used further in quantitative structure-retention relationship (QSRR) studies in order to establish predictive models that correlate molecular structure with chromatographic behavior and to reveal which molecular features are responsible for specific retention behaviors.

The present study is focused on experimental characterization of chromatographic behavior and lipophilicity of two groups of alkylaminoethyl androstane-3-oxime derivatives, including 17β-hydroxy-17α-(pyridin-2-ylmethyl)androst-4-en-(3*E*,*Z*)-one oximes and (17*E*)-(pyridin-2-ylmethylidene)androst-4-en-(3*E,Z*)-one oximes, as compounds with significant anticancer activity [12,17]. The analysis is performed in reversed-phase ultra-high performance liquid chromatography (RP-UHPLC) systems on three different stationary phases (C8, C18 and phenyl), applying two binary (methanol/water and acetonitrile/water) and one ternary mobile phase (methanol/acetonitrile/water). The obtained chromatographic lipophilicity parameters characterize the retention behavior of the synthesized androstane derivatives across hydrophobic and π-interacting environments. The parameters were further analyzed, applying chemometric methods including linear and non-linear pattern recognition techniques. The obtained parameters provide an assessment of molecular lipophilicity, reflecting not only the compound’s affinity for non-polar phases but also its responsiveness to changes in mobile phase composition and stationary phase chemistry. These data serve as a base for further QSRR modeling and offer valuable insight into the molecular features governing retention behavior and molecular lipophilicity.

## 2. Results

### 2.1. Lipophilicity Determination on C18 Column

The C18 column is a widely used stationary phase for determination of chromatographic lipophilicity of various compounds [18] since it possesses long hydrophobic chains that provide strong non-polar interactions, which is particularly suitable for characterization of lipophilic compounds. Both groups of analyzed *O*-alkylated androstane-3-oxime derivatives were analyzed by using defined chromatographic conditions on a C18 phase. The obtained values of chromatographic parameters (log*k*) are presented in Table 1. The determined parameters indicate that all the compounds exhibited the highest retention in the system with methanol as a modifier and the lowest retention when acetonitrile was applied as a modifier.

The in silico lipophilicity parameters of the analyzed compounds are provided in Table 2.

**Figure 1:**
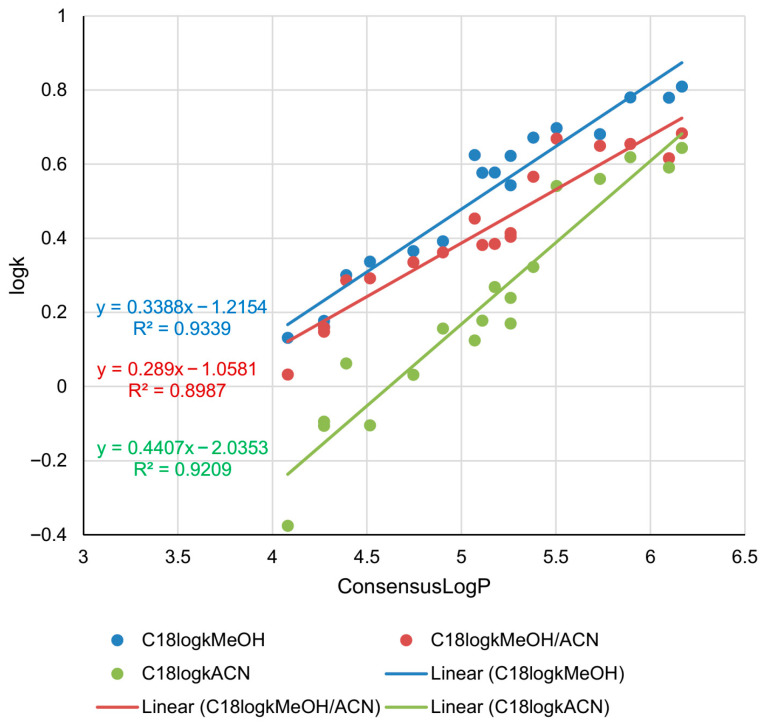
Correlations between in silico lipophilicity and retention parameters (log*k*) of the analyzed androstane-3-oxime derivatives determined in the RP-UHPLC system with C18 stationary phase and three different mobile phases.

The relationships between the experimental log*k* values obtained by using C18 column and in silico lipophilicity measures (log*P*) were analyzed. The obtained linear relationships are presented in Figure 1. As can be seen, the established relationships are described by high determination coefficients for all three mobile phases. The best fit of the experimental and in silico data was achieved in the system with methanol as a modifier (*R*^2^ = 0.9339). The system with a ternary mobile phase had the lowest determination coefficient (*R*^2^ = 0.8987).

### 2.2. Lipophilicity Determination on C8 Column

Octyl columns are moderately lipophilic, so lipophilic compounds are retained to a lesser extent on octyl than on octadecyl columns [19]. Shorter carbon-alkyl chains provide weaker hydrophobic interactions than on C18 columns. Taking into account the fact that the studied derivatives are considered to be lipophilic to highly lipophilic (based on in silico log*P* values), although octyl columns are perhaps at first glance unsuitable for the analysis, the application of C8 columns to test their experimental lipophilicity profile provided comparative profiling of their chromatographic behavior. The obtained capacity factors are presented in Table 1. These results indicate significantly lower retention of the analyzed compounds on the C8 phase than on the C18 phase. Considering the retention of (*E*)-OH and (*Z*)-OH isomers (compounds **1** and **2**, and **9** and **10**), the same chromatographic behavior is observable as in the system with C18 phase.

**Figure 2:**
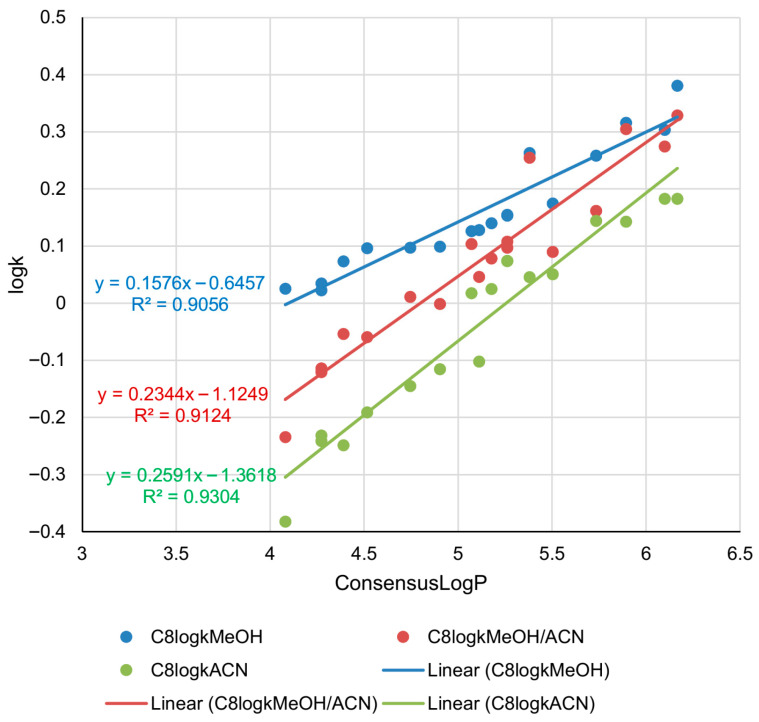
Correlations between in silico lipophilicity and retention parameters (log*k*) of the analyzed androstane-3-oxime derivatives determined in RP-UHPLC system with C8 stationary phase and three different mobile phases.

The retention parameters determined on the C8 column in all three mobile phases, presented in Table 1, were further correlated with in silico lipophilicity (ConsensusLogP). The established linear relationships are presented in Figure 2.

### 2.3. Lipophilicity Determination on Phenyl Column

**Figure 3:**
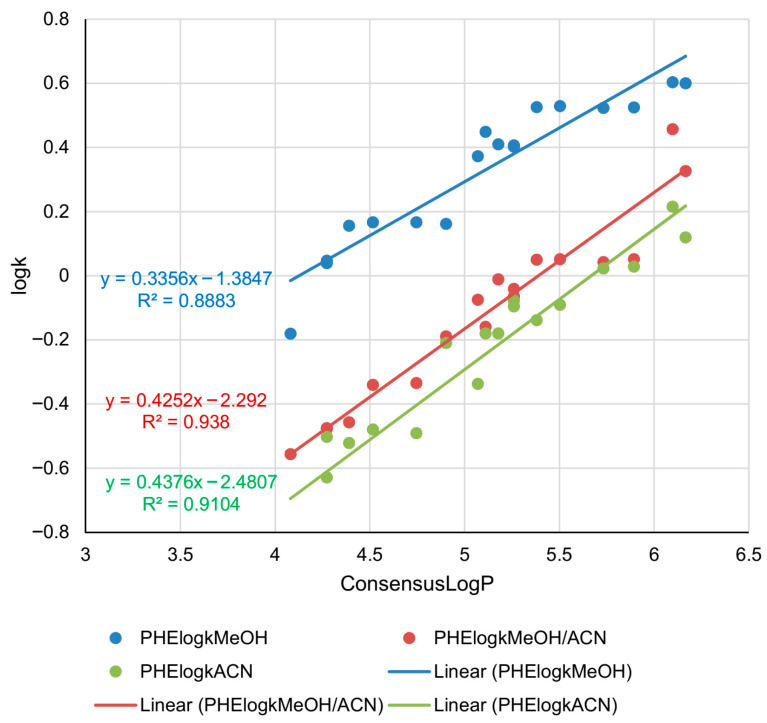
Correlations between in silico lipophilicity and retention parameters (log*k*) of the analyzed androstane-3-oxime derivatives determined in RP-UHPLC system with phenyl stationary phase and three different mobile phases.

Phenyl columns are not a standard choice for lipophilicity estimation; however, they can provide useful information regarding the chromatographic behavior of compounds with aromatic systems. π–π interactions (π-stacking) with aromatic systems are typical for phenyl columns, in addition to hydrophobic interactions. All the studied compounds possess aromatic rings, so π-stacking is expected to occur during the chromatographic analysis of all the compounds. The data provided in Table 1 indicate that the retention of the (3*Z*)-NOH isomer (compound **2**) is higher than the retention of the (3*E*)-NOH isomer (compound **1**), unlike the retention on C18 and C8 columns. In the mobile phases with methanol/acetonitrile and acetonitrile, the (3*E*)-NOH isomer has higher retention than the (3*Z*)-NOH isomer. Considering isomers **9** and **10**, it can be seen that they behave in the system with the phenyl column in the same manner as on C8 and C18 columns. The established linear relationships between ConsensusLogP and log*k* parameters obtained on phenyl column are given in Figure 3.

### 2.4. Chemometrics of the Experimental Lipophilicity Data

**Figure 4:**
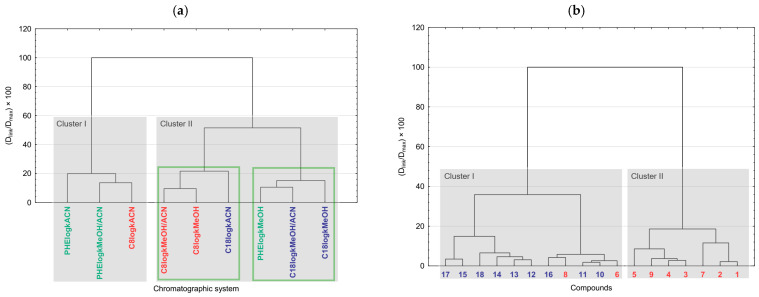
Dendrogram of the clustering of (**a**) chromatographic systems and (**b**) androstane-3-oxime derivatives.

Chemometric pattern recognition analysis was applied in order to gain an overview of the similarities and dissimilarities among the analyzed androstane-3-oxime derivatives in the space of determined chromatographic lipophilicity data. The dendrograms are presented in Figure 4.

**Figure 5:**
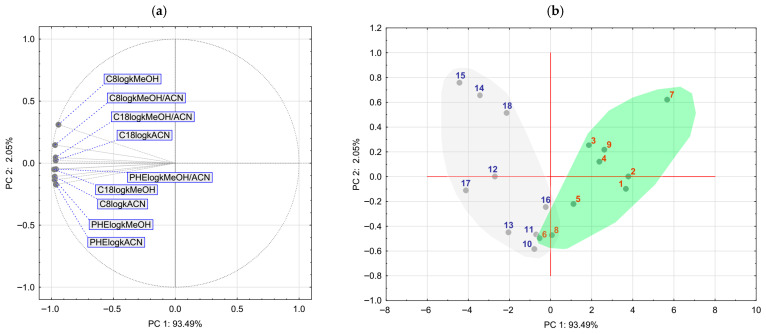
Loadings (**a**) and score (**b**) plot of the results of PCA.

PCA was applied in order to reveal underlying variance structure and distribution of the compounds in the space of principal components. The resulting loadings and score plots are presented in Figure 5.

**Figure 6:**
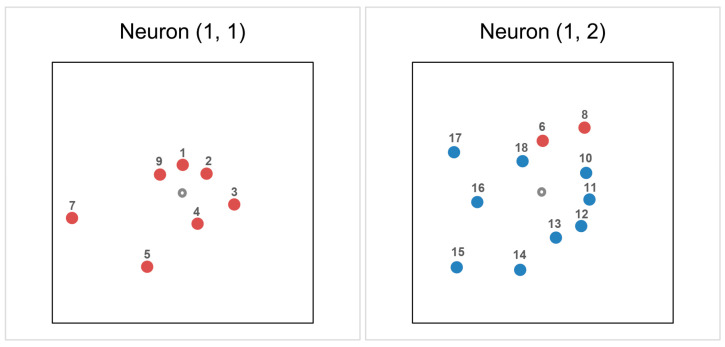
Kohonen graph of results of CANN analysis: two neurons in the topological lattice (1, 1) and (1, 2) and the compounds grouped in the neurons.

Although HCA and PCA revealed consistent groupings, ANN-based clustering was performed in order to provide non-linear validation and to confirm the robustness of the clusters. The Kohonen graph shown in Figure 6 presents the results of CANN analysis of the studied androstane-3-oxime derivatives based on the same input data as in HCA and PCA. Each box is a neuron in the topological lattice. The division of the compounds in the training, test and validation set, as well as neuron identification and activation, are presented in Table 3. A small activation value means the shortest Euclidean distance to the data case.

## 3. Discussion

By comparing the retention of (3*E*)-NOH and (3*Z*)-NOH isomers in the C18 system (the pairs **1** and **2**, as well as **9** and **10**) it can be noticed that both (3*E*)-NOH isomers have higher retention than (3*Z*)-NOH isomers in the mobile phase with methanol. However, in the ternary mobile phase, the isomer from the 17-picolyl series (compound **2** as (3*Z*)-NOH isomer) has higher retention than the corresponding isomer (compound **1** as (3*E*)-NOH isomer). This is also observable when the mobile phase with acetonitrile is applied. The (3*Z*)-NOH isomer (compound **11**) from the picolinylidene series has higher retention than the corresponding (3*E*)-NOH isomer (compound **10**). These slight differences between the retention of the aforementioned isomers exist primarily due to variations in intramolecular interactions, molecular shape, steric effects and the potential influence of isomerism on total molecular polarity.

Considering all three C18 systems with different modifiers, it can be seen that the compounds from the17-picolynilidene series have higher retention than the compounds from the 17-picolyl series, probably due to the higher lipophilicity of the 17-picolinylidene moiety than the 17β-hydroxy-17α-picolyl moiety.

The obtained results indicate that determined retention parameters can be considered very good experimental lipophilicity measures of the examined compounds. Those anisotropic lipophilicity parameters reflect the dependence of molecular interactions within the chromatographic system [20] and particularly those governed by hydrophobic partitioning, dipole–dipole interactions and steric accessibility between analytes and the stationary phase. Also, the variations in retention behavior in the applied chromatographic systems can also be explained by the fact that methanol is a protic solvent, while acetonitrile is a typical aprotic solvent. This fact is particularly important since methanol can donate H-bonds and enhance the interaction with polar analytes, stabilizing the interactions between the analyte and stationary phase chains and leading to longer retention. On the other hand, acetonitrile has limited capacity for specific interactions with polar analytes due to its aprotic nature. As a mobile phase modifier, it provides faster elution and reduces retention of polar analytes. In the case of ternary mobile phase, the interactions between the analyte, mobile phase components and stationary phase become more complex. Which interactions will be dominant depends on the volumes of mobile phase components. Those interactions include hydrophobic interactions, hydrogen bonding and dipole–dipole interactions, all of which are influenced by the polarity and hydrogen-bonding capacity of the applied organic solvents. It can be concluded that the retention of the analyzed *O*-alkylated androstane-3-oxime derivatives is extreme in binary mobile phases, while in the case of the ternary mobile phase, it is moderate. Taking into account the slopes of the relationships presented in Figure 1 for the system with methanol (blue trendline) and the system with methanol/acetonitrile mobile phase (red trendline), it can be noticed that retention of highly lipophilic compounds differs more in these two systems than the retention of compounds with moderate lipophilicity. The opposite observation is true for comparison of the retention determined by using mobile phase with methanol/acetonitrile (red trendline) and the mobile phase with acetonitrile (green trendline).

The relationship describing the C8 system with methanol is described by slightly a lower determination coefficient (*R*^2^ = 0.9056) than in the case of the C18 system with the same modifier (*R*^2^ = 0.9339). Also, the slope of the ConsensusLogP-log*k* relationship in the C8 system is twice as small as in the C18 system, meaning that minor differences in lipophilicity result in significant changes in retention, and consequently, in the chromatographic behavior of the C18 system compared to the C8 system. The retention of highly lipophilic compounds is quite similar in the system with methanol and the system with methanol/acetonitrile (the blue and red trendlines intersect at lipophilicity values higher than 6). In the C8 system, the best fitting of the data was achieved with acetonitrile as a modifier (*R*^2^ = 0.9304), therefore log*k* values determined under those conditions are better representatives of lipophilicity of the analyzed compounds than in the ternary mixture and mixture with methanol. Generally, based on the obtained relationships, it can be concluded that the retention parameters obtained by using the C8 stationary phase are good predictors of the lipophilicity of the analyzed derivatives.

Considering the system with the phenyl column, the retention of all tested compounds is the lowest in the mobile phase with acetonitrile, slightly higher in the mobile phase with methanol/acetonitrile, and the highest in the mobile phase with only methanol as a modifier. In Figure 3, it can be noticed that a drop in retention of all compounds is evident when the mobile phases with acetonitrile were used. The introduction of acetonitrile as an aprotic solvent in the mobile phase led to faster elution of the analytes due to stronger hydrophobic interactions with mobile phase. Considering the fact that all three trendlines are parallel, it can be assumed that the relative retention behavior of compounds based on lipophilicity is preserved, regardless of mobile phase composition, as well as consistent selectivity for lipophilicity across different mobile phase compositions. The strongest ConsensusLogP-log*k* relationship is expressed in the system with the ternary mobile phase (red trendline, *R*^2^ = 0.938). The lowest determination coefficient (*R*^2^ = 0.8883) and the lowest slope (0.3356) are related to the system with methanol as a modifier (blue trendline). The chromatographic parameters determined on a phenyl column can be considered a good lipophilicity measure of the studied derivatives considering high correlations between the ConsensusLogP and log*k* parameters. Although retention shifts with mobile phase polarity (acetonitrile is less polar than methanol, but in RP-UHPLC, it behaves as a stronger eluent), there is a consistent linear dependence between retention and lipophilicity.

**Figure 7:**
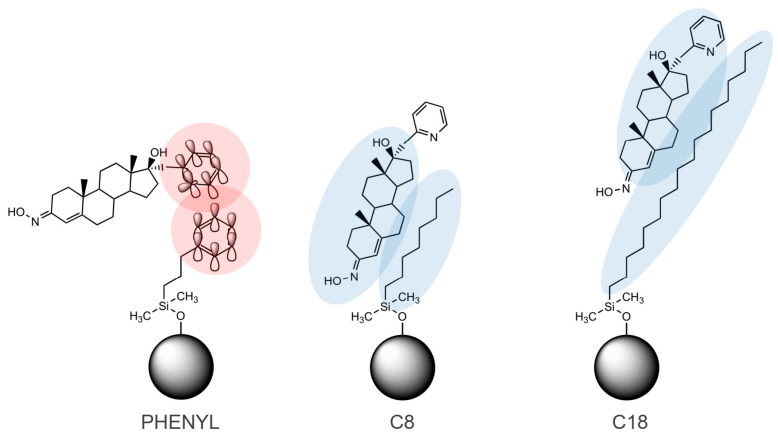
Primary interactions between a steroidal analyte and different stationary phases: π–π (red) and hydrophobic (blue) interactions.

Comparative analysis implies that the application of three different stationary phases and three mobile phases enables a multidimensional assessment of lipophilicity by capturing both hydrophobic and π–π interactions. This approach enhances the reliability of log*P*-log*k* correlations and validates the assumption that the lipophilicity of the studied androstane-3-oximes remains the dominant factor across chromatographic conditions. The main intermolecular interactions between compound **1**, as an example, and applied stationary phases are illustrated in Figure 7. Hydrophobic interactions are dominant across all three stationary phases; however, π–π interactions are also typical for the phenyl column and influence slightly different chromatographic behaviors than in the case of stationary phases with alkyl chains. Considering the high portion of modifiers in the mobile phase (0.8 *v*/*v*), their influence on retention is quite significant.

**Figure 8:**
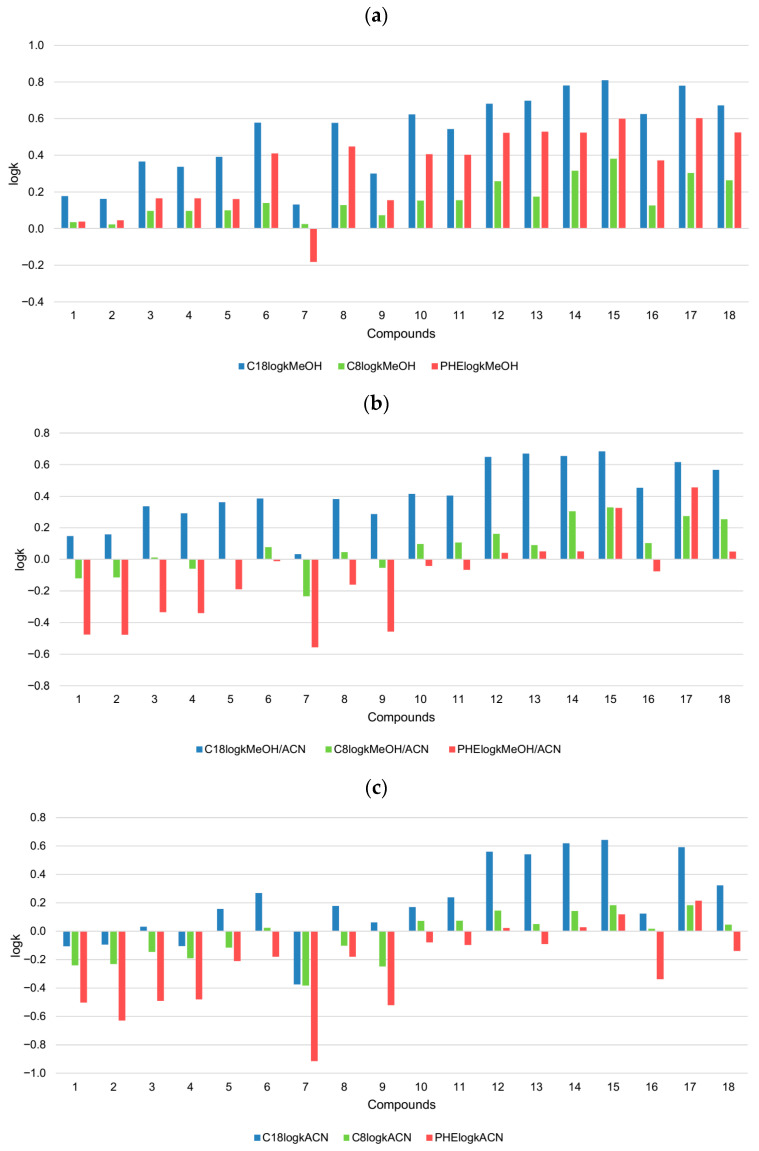
Comparative analysis of the chromatographic behavior of androstane-3-oxime derivatives on different columns in the same mobile phase with the following modifiers: (**a**) methanol, (**b**) methanol/acetonitrile and (**c**) acetonitrile.

A comparative analysis of chromatographic lipophilicity of the analyzed androstane-3-oxime derivatives across the C18, C8 and phenyl stationary phases and three mobile phases is graphically presented in Figure 8.

When the methanol/water mixture is used as a mobile phase (Figure 8a), the highest retention of the majority of compounds is achieved on the C18 stationary phase, while the lowest retention can be observed with the C8 phase. Generally, the observed decreasing pattern in retention for the methanol/water mixture is as follows:

C18 > phenyl > C8

However, compound **7** is considered to be the exception, since it has the lowest retention on the phenyl phase and the lowest lipophilicity.

Also, it can be observed that 17β-hydroxy-17α-picolyl derivatives have generally lower retention than 17-picolinylidene derivatives under applied chromatographic conditions. The reason for this is probably the fact that the 17β-hydroxy-17α-picolyl system has a lower lipophilicity than the (17*E*)-picolinylidene system.

When an equivolume mixture of methanol and acetonitrile is applied as a modifier, the retention of the compounds significantly decreases (Figure 8b). The highest change is observable on the phenyl column. The reason for this could be the combined enhanced elution strength of the solvent and its ability to suppress π–π interactions more effectively than methanol alone. This suppression is less expressed in the case of some compounds that are highly lipophilic, such as compounds **15** and **17**. Retention behavior in the methanol/acetonitrile/water and acetonitrile/water systems follows this general decreasing trend:

C18 > C8 > phenyl

The mobile phase with acetonitrile (Figure 8c) significantly decreased the retention, particularly for 17β-hydroxy-17α-picolyl derivatives.

HCA was applied and two dendrograms were obtained. The dendrogram presented in Figure 4a depicts the clustering of the chromatographic systems. There are two main clusters observable. Cluster I contains three chromatographic systems, including two systems with phenyl column with acetonitrile and acetonitrile/water mixture, and the system with the C8 column with acetonitrile. The other systems are placed in cluster II and divided into two sub-clusters, indicating similar retention behavior of the compounds in the following systems: C8log*k*MeOH, C8log*k*MeOH/ACN, and C18log*k*ACN, as well as C18log*k*MeOH, C18log*k*MeOH/ACN, and PHElog*k*MeOH. In Figure 4b, there is a dendrogram of the clustering of the analyzed compounds. This dendrogram indicates a general, but not strict, separation between 17β-hydroxy-17α-picolyl and (17*E*)-picolinylidene derivatives. However, cluster I contains compounds **6** and **8** (picolyl series) among the 17-picolinylidene derivatives. This implies that the retention behavior of these two derivatives is much closer to 17-picolinylidene derivatives than to the rest of the 17-picolyl compounds. Also, in the 17-picolyl series, compounds **6** and **8** have the highest lipophilicity.

PCA provided a loading plot (Figure 5a) that indicates a dominant influence of all considered variables on the negative end of the PC1 axis. On the other hand, the C8log*k*MeOH variable had a moderate influence towards the positive end of the PC2 axis. Therefore, the most significant grouping of the compounds is expected to be on the PC1 axis. As it can be seen on the score plot (Figure 5b), the two groups of compounds (17-picolyl and 17-picolinylidene derivatives) can be clearly distinguished. There is a certain overlap observable: compounds **6** and **8** are placed quite close to the 17-picolinylidene group, as in the case of HCA, in which these two compounds are placed in the cluster with 17-picolinylidene derivatives. The PCA reveals that compounds **14** and **15** can be distinguished from other derivatives based on the C8log*k*MeOH variable: these compounds have significantly higher retention in the chromatographic system than other compounds.

The results of CANN analysis clearly indicate the separation of the compounds into two clusters (two neurons). In the first neuron, there are only the compounds from the 17-picolyl series (without compounds **6** and **8**), while the compounds from the 17-picolinylidene series are located in the second neuron, in which the compounds **6** and **8** are placed as well. This fact is another confirmation that compounds **6** and **8** are closer to the group of 17-picolinylidene derivatives based on their retention behavior in the applied RP-UHPLC systems. This convergence of clustering outcomes across HCA, PCA and CANN reveals a robust underlying data structure and confirms that both the similarities and dissimilarities among the analyzed derivatives, in terms of their chromatographic lipophilicity, are stable and reproducible, regardless of the pattern recognition approach used.

## 4. Materials and Methods

### 4.1. Androstane-3-Oxime Derivatives

The synthesis of the analyzed compounds was performed at the Department of Chemistry, Biochemistry and Environmental Protection, Faculty of Sciences, University of Novi Sad [12]. The molecular structures are presented in Table 4 and they are divided into two main groups: the group A of nine 17β-hydroxy-17α-(pyridin-2-ylmethyl)androst-4-en-(3*E*,*Z*)-one oximes and the group B of nine (17*E*)-(pyridin-2-ylmethylidene)androst-4-en-(3*E,Z*)-one oximes.

### 4.2. UHPLC Chromatographic Analysis

The experimental determination of lipophilicity was performed on the Agilent 1290 Infinity LC system with Diode Array Detector on three types of columns: (1) ZORBAX Extend-C18, Rapid Resolution HT, 2.1 × 100 mm, 1.8 µm (600 bar pressure limit), (2) ZORBAX Eclipse SB-C8, Rapid Resolution HT, 2.1 × 100 mm, 1.8 µm (600 bar pressure limit) and (3) ZORBAX Eclipse XDB-Phenyl, 2.1 × 150 mm, 5 µm (400 bar pressure limit). The prepared compounds were dissolved in acetone (HPLC-grade, Carlo Erba, Milan, Italy) at a concentration of 1 mg/mL and subsequently filtered using Captiva Econofilter equipped with a nylon membrane (25 mm diameter, 0.45 µm pore size, 1000/pk). A 10 µL aliquot of the sample was injected for analysis. The chromatographic procedure was conducted under isocratic conditions, with the column temperature controlled at 25 °C and a flow rate of 0.2 mL/min for the C18 and C8 columns and 0.5 mL/min for the phenyl column. The analysis was carried out in triplicate. The mobile phases utilized consisted of binary or ternary mixtures comprising methanol (HPLC gradient grade, J.T.Baker, Deventer, The Netherlands), acetonitrile (HPLC-grade, Acros Organics, Geel, Belgium) and distilled water (HPLC grade). The specific compositions were as follows:

(1) Mobile phase A: methanol/water (protic modifier);
(2) Mobile phase B: methanol/acetonitrile/water (combination of protic and aprotic modifiers);
(3) Mobile phase C: acetonitrile/water (aprotic modifier).

The modifiers in the mixtures were included at a volume fraction of 0.8 *v*/*v*. In mobile phase B, the individual modifiers were present in equal volumes. Chromatographic peaks were detected at a wavelength of 210 nm. The chromatographic parameters, specifically the capacity factor (*k*), were determined using the retention time of the compound’s peak (*t_r_*) and the retention time of the solvent peak (*t*_0_), commonly referred to as dead time, typically observed as the first disturbance on the chromatogram:

*k* = (*t_r_* − *t*_0_)/*t*_0_ (1)

The logarithmic form of the capacity factor (log*k*) was utilized in subsequent analyses.

### 4.3. In Silico Lipophilicity Descriptors

In silico lipophilicity descriptors were calculated based on 2D structures or based on the SMILES codes of the analyzed compounds. The calculations were performed by using different programs: WLOGP was calculated by SWISSADME online software, VGlogP, KLOPlogP, PHYSlogP and WGlogP by applying the MarvinSketch v.14.9.15.0 program and logPCD by using the ChemBioDraw Ultra 13.0 program. The consensus LogP parameter (ConsensusLogP) was calculated as the average of all obtained logP parameters.

### 4.4. Chemometric Analysis

Hierarchical cluster analysis (HCA) and principal component analysis (PCA) were performed applying Statistica v.12 software. HCA was based on Ward’s algorithm as a linkage rule. Euclidean distances were used as a distance measure. The values on the *y*-axis were normalized using the equation D_link_/D_max_ × 100, where D_link_ represents the linkage distance at which two clusters are merged, highlighting the degree of dissimilarity at the point of fusion, while D_max_ denotes the highest dissimilarity observed among any pair of clusters. This normalization expresses linkage distances as percentages relative to the maximum observed value. PCA was performed based on correlation matrices, with variances calculated using the formula *SS*/(*N* − 1), where *SS* is the sum of squares and *N* is the number of samples. Cluster analysis via artificial neural networks (CANN) employed Kohonen training to uncover inherent data structures. The dataset was partitioned into 70% for training, 15% for testing, and 15% for validation. The topological map was configured with a height of 2 and a width of 3, and the training process encompassed 5000 cycles. Pattern recognition analysis was performed on natural data, since all the variables were on the same scale; therefore, normalization of the data was not necessary.

## 5. Conclusions

The chromatographic profiling of adrostane-3-oxime derivatives across diverse RP-UHPLC systems with different stationary and mobile phases enabled a comprehensive assessment of their anisotropic lipophilicity. The derived log*k* values can be considered reliable lipophilicity measures of the analyzed compounds, considering their high correlations with in silico log*P* parameters. The obtained log*k* values were interpreted through both linear (HCA, PCA) and non-linear (CANN) pattern recognition techniques, and consistent clustering patterns among the molecules were observed. The comparative analysis showed that the group of 17β-hydroxy-17α-picolyl androstane-3-oxime derivatives possesses general lower experimental lipophilicity (lower chromatographic retention) than (17*E*)-picolinylidene derivatives. These findings not only define the physicochemical properties of the studied compounds but also establish a robust analytical framework for future investigations into lipophilicity–bioactivity relationships.

## Figures and Tables

**Table 1:** Chromatographic parameters (log*k*) of the analyzed androstane-3-oxime derivatives in the applied UHPLC system with three different mobile phases and three chromatographic columns.

Compound	C18log*k*MeOH	C18log*k*MeOH/ACN	C18log*k*ACN	C8log*k*MeOH	C8log*k*MeOH/ACN	C8log*k*ACN	PHElog*k*MeOH	PHElog*k*MeOH/ACN	PHElog*k*ACN
**1**	0.178	0.148	−0.106	0.035	−0.121	−0.241	0.039	−0.475	−0.502
**2**	0.162	0.159	−0.095	0.023	−0.114	−0.231	0.046	−0.476	−0.629
**3**	0.366	0.336	0.032	0.097	0.011	−0.145	0.166	−0.335	−0.491
**4**	0.338	0.292	−0.104	0.097	−0.059	−0.191	0.166	−0.341	−0.480
**5**	0.392	0.362	0.157	0.099	−0.001	−0.116	0.161	−0.190	−0.210
**6**	0.578	0.385	0.269	0.140	0.079	0.025	0.409	−0.011	−0.180
**7**	0.132	0.033	−0.376	0.025	−0.234	−0.382	−0.181	−0.557	−0.915
**8**	0.577	0.383	0.178	0.128	0.046	−0.102	0.448	−0.160	−0.180
**9**	0.301	0.287	0.062	0.073	−0.054	−0.248	0.155	−0.457	−0.522
**10**	0.623	0.414	0.171	0.154	0.097	0.074	0.406	−0.041	−0.078
**11**	0.544	0.405	0.239	0.155	0.108	0.074	0.402	−0.065	−0.096
**12**	0.681	0.650	0.561	0.258	0.162	0.144	0.522	0.042	0.022
**13**	0.698	0.669	0.542	0.175	0.090	0.051	0.529	0.051	−0.091
**14**	0.780	0.655	0.619	0.316	0.305	0.143	0.524	0.051	0.028
**15**	0.810	0.683	0.644	0.381	0.329	0.183	0.599	0.326	0.119
**16**	0.625	0.453	0.125	0.126	0.104	0.018	0.373	−0.075	−0.337
**17**	0.780	0.616	0.591	0.304	0.275	0.183	0.603	0.456	0.215
**18**	0.672	0.566	0.323	0.263	0.255	0.046	0.525	0.050	−0.139

Modifiers: MeOH—methanol, ACN—acetonitrile, MeOH/ACN—equivolume methanol and acetonitrile mixture.

**Table 2:** In silico lipophilicity parameters of androstane-3-oxime derivatives.

Compound	WLOGP	VGlogP	KLOPlogP	PHYSlogP	WGlogP	logPCD	ConsensusLogP
**1**	5.150	3.500	4.020	4.555	4.201	4.210	4.273
**2**	5.150	3.500	4.020	4.555	4.201	4.210	4.273
**3**	5.410	4.722	4.224	4.754	4.572	4.790	4.745
**4**	5.640	4.449	3.819	4.394	4.212	4.580	4.516
**5**	5.790	4.845	4.305	4.909	4.697	4.870	4.903
**6**	5.800	5.118	4.694	5.222	5.028	5.200	5.177
**7**	4.640	4.054	3.686	4.087	3.948	4.070	4.081
**8**	6.030	5.082	4.534	5.008	4.862	5.150	5.111
**9**	5.250	4.396	3.806	4.279	4.141	4.470	4.390
**10**	6.150	4.900	4.733	5.563	5.176	5.040	5.260
**11**	6.150	4.900	4.733	5.563	5.176	5.040	5.260
**12**	6.410	6.121	4.938	5.762	5.547	5.620	5.733
**13**	6.640	5.848	4.533	5.402	5.187	5.410	5.503
**14**	6.800	6.244	5.019	5.917	5.672	5.710	5.894
**15**	6.800	6.517	5.407	6.230	6.003	6.040	6.166
**16**	5.640	5.453	4.400	5.095	4.923	4.910	5.070
**17**	7.030	6.480	5.247	6.016	5.837	5.980	6.098
**18**	6.250	5.796	4.520	5.288	5.116	5.310	5.380

**Table 3:** Comparative analysis of the chromatographic behavior of androstane-3-oxime derivatives in the same mobile phase on (a) C18 column, (b) C8 column and (c) phenyl column.

Compound	Set	Neuron No.	Position	Activations
**1**	Train	1	(1, 1)	0.257
**2**	Train	1	(1, 1)	0.281
**3**	Validation	1	(1, 1)	0.486
**4**	Train	1	(1, 1)	0.316
**5**	Validation	1	(1, 1)	0.756
**6**	Train	2	(1, 2)	0.476
**7**	Test	1	(1, 1)	1.045
**8**	Train	2	(1, 2)	0.712
**9**	Train	1	(1, 1)	0.267
**10**	Train	2	(1, 2)	0.438
**11**	Train	2	(1, 2)	0.434
**12**	Train	2	(1, 2)	0.468
**13**	Train	2	(1, 2)	0.430
**14**	Train	2	(1, 2)	0.739
**15**	Test	2	(1, 2)	1.049
**16**	Train	2	(1, 2)	0.609
**17**	Train	2	(1, 2)	0.899
**18**	Train	2	(1, 2)	0.345

**Table 4:** The molecular structures of the analyzed androstane-3-oxime derivatives.

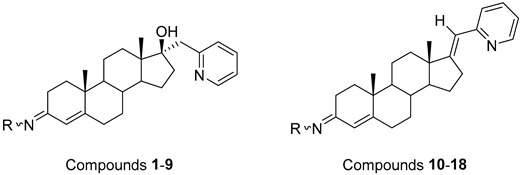		
Compounds of GROUP A (17-Picolyl Series)	R	Compounds of GROUP B (17-Picolynilidene Series)
**1**	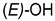	**10**
**2**	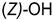	**11**
**3**	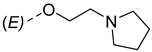	**12**
**4**	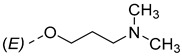	**13**
**5**	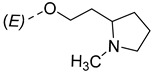	**14**
**6**	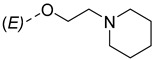	**15**
**7**	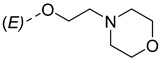	**16**
**8**	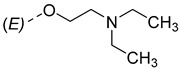	**17**
**9**	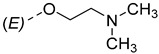	**18**

## Data Availability

The original contributions presented in this study are included in the article. Further inquiries can be directed to the corresponding author.

## Abbreviations

The following abbreviations are used in this manuscript:

RP	Reversed-Phase
UHPLC	Ultra-High Performance Liquid Chromatography
PRT	Pattern Recognition Techniques
CANN	Clustering by Artificial Neural Networks
HCA	Hierarchical Cluster Analysis
PCA	Principal Component Analysis
MeOH	Methanol
ACN	Acetonitrile
